# Molecular Profiling of Pierce’s Disease Outlines the Response Circuitry of *Vitis vinifera* to *Xylella fastidiosa* Infection

**DOI:** 10.3389/fpls.2018.00771

**Published:** 2018-06-08

**Authors:** Paulo A. Zaini, Rafael Nascimento, Hossein Gouran, Dario Cantu, Sandeep Chakraborty, My Phu, Luiz R. Goulart, Abhaya M. Dandekar

**Affiliations:** ^1^Department of Plant Sciences, University of California, Davis, Davis, CA, United States; ^2^Institute of Genetics and Biochemistry, Federal University of Uberlândia, Uberlândia, Brazil; ^3^Department of Viticulture and Enology, University of California, Davis, Davis, CA, United States

**Keywords:** defense response, Xanthomonadaceae, plant–bacteria interaction, vascular pathogen, transcriptome, proteome, metabolome

## Abstract

Pierce’s disease is a major threat to grapevines caused by the bacterium *Xylella fastidiosa*. Although devoid of a type 3 secretion system commonly employed by bacterial pathogens to deliver effectors inside host cells, this pathogen is able to influence host parenchymal cells from the xylem lumen by secreting a battery of hydrolytic enzymes. Defining the cellular and biochemical changes induced during disease can foster the development of novel therapeutic strategies aimed at reducing the pathogen fitness and increasing plant health. To this end, we investigated the transcriptional, proteomic, and metabolomic responses of diseased *Vitis vinifera* compared to healthy plants. We found that several antioxidant strategies were induced, including the accumulation of gamma-aminobutyric acid (GABA) and polyamine metabolism, as well as iron and copper chelation, but these were insufficient to protect the plant from chronic oxidative stress and disease symptom development. Notable upregulation of phytoalexins, pathogenesis-related proteins, and various aromatic acid metabolites was part of the host responses observed. Moreover, upregulation of various cell wall modification enzymes followed the proliferation of the pathogen within xylem vessels, consistent with the intensive thickening of vessels’ secondary walls observed by magnetic resonance imaging. By interpreting the molecular profile changes taking place in symptomatic tissues, we report a set of molecular markers that can be further explored to aid in disease detection, breeding for resistance, and developing therapeutics.

## Introduction

Plants have evolved complex responses to adapt to both biotic and abiotic environmental stresses, with an increase in the production of reactive oxygen species (ROS) as a key mechanism common to several types of stress conditions (Mittler, 2017). Genome sequencing and other high-throughput approaches have greatly advanced the identification of specific responses, enabling the classification of hundreds of genes responsive to particular stresses. In *Vitis vinifera* for example, different abiotic and biotic stressors have been studied (Lin et al., 2007; Choi et al., 2013; Abou-Mansour et al., 2015; Burger and Maree, 2015; Czemmel et al., 2015; Dadakova et al., 2015; Kelloniemi et al., 2015; Savoi et al., 2016), which have provided important insights on the molecular aspects of Pierce’s disease (PD) development following infection by the bacterium *Xylella fastidiosa*. The disease can be transmitted by infected plant material and tools, as well as by many species of xylem sap-feeding insects that vector the pathogen from plant to plant (Chatterjee et al., 2008). Detection and spread of different strains in the European continent in recent years have raised great concern, given its potential to colonize plant hosts already attacked in the American continent such as grapevines and oranges among others. It also poses the threat of causing new diseases like olive quick decline syndrome, which is annihilating olive groves in southern Italy and progressively spreading in the Mediterranean area (Martelli et al., 2016). Disease progression varies widely depending on environmental conditions and genotypes of pathogen and host scion and rootstocks, collectively contributing to different chemical microenvironments of scion sap (Wallis et al., 2013; Katam et al., 2015). Successful microbe proliferation and long distance movement across xylem elements are mediated by secreted virulence factors such as the polygalacturonase PglA, the lipase/esterase LesA, and protease PrtA that are able to modify xylem integrity by enzymatic activity (Agüero et al., 2005; Roper et al., 2007; Gouran et al., 2016; Nascimento et al., 2016), and together contribute to disease progression. Vascular occlusions caused by pathogen biofilm formation, host cell-wall thickening by callose deposition and lignification, as well as tylose formation all contribute to reduction of sap flow leading to water and nutrient limitation as symptoms progress (Chatterjee et al., 2008; Choi et al., 2013; Sun et al., 2013). Despite significant advances, however, the grapevine response circuitry to PD is still not thoroughly characterized. Here we report a systems biology approach to expand our understanding of the cellular and molecular changes during PD development in Thompson seedless grapevines. Our data highlighted major metabolites accumulated in PD and also revealed novel members of the pathogen-sensing and stress response network. This enabled us to select which genes within paralog groups play a more pronounced role in the defense response to PD and thus can be further explored as early disease markers or therapeutic targets in case of disease susceptibility genes. Since current mitigation strategies to control PD rely on intensive insecticide applications to prevent vectors from disseminating *X. fastidiosa* across grapevines, understanding disease susceptibility and the host molecular responses to infection can lead to improved resistance breeding and novel control approaches.

## Materials and Methods

### *V. vinifera* Inoculation With *X. fastidiosa* and Preparation of Leaf Extracts

Controlled inoculations of 3-month-old clonally propagated grapevines (*V. vinifera* var. Thompson Seedless) were performed according to (Dandekar et al., 2012). Briefly, plants were laid horizontally and 10 μL of succinate-citrate buffer containing ∼10^6^ cells of *X. fastidiosa* strain Temecula1 (NCBI Accession PRJNA285) was deposited on the cane ∼10 cm above soil level and punctured with a needle to allow for uptake of bacterial suspension into the xylem. Plants were kept in greenhouse conditions (20–25°C and watered daily) and leaf samples collected 12 weeks post inoculations. Preparation of grapevine leaf extracts was done as described in Nascimento et al. (2016) using three complete leaves excluding petioles from eight infected and eight control (inoculated with succinate-citrate buffer without *X. fastidiosa*) plants. Briefly leaves were flash frozen in liquid nitrogen, ground with mortar and pestle, and kept at -80°C until use.

### Nuclear Magnetic Resonance Imaging

**FIGURE 1:**
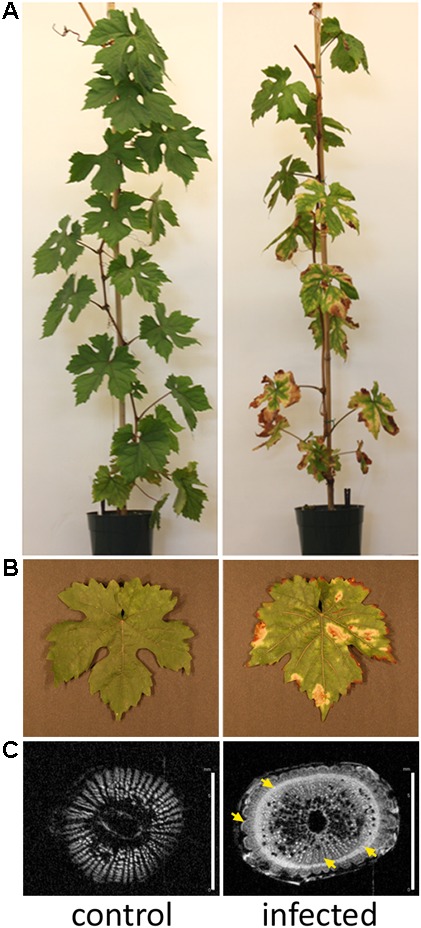
Grapevines developing Pierce’s disease in controlled inoculations. **(A)** Twelve weeks after inoculation of *X. fastidiosa*, grapevines already displayed Pierce’s disease symptoms as leaf scorching, match stick petioles from fallen leaves, and darkened patches on stems. **(B)** Detail of leaf showing scorching symptoms. **(C)** Nuclear magnetic resonance imaging of representative transversal cuts of stems exhibiting intensive secondary wall deposition in infected vines. Yellow arrows point to electron-denser material more abundant in infected samples. Scale bar is 6 mm for both images.

Nuclear magnetic resonance imaging (^1^H-MRI) was done in an Avance 400 spectrometer equipped with Bruker DRX console microimaging accessory according to (Dandekar et al., 2012). Stem transverse sections of all non-infected and infected plants were collected between internodes located at the top (apical), middle, and bottom of the central stem (three cuts per plant) and subjected to MRI. **Figure 1C** shows representative results.

### RNA Extraction, Library Preparation, and Sequencing

RNA extraction from 1 g of ground leaf tissue was done with MasterPure Complete kit (Epicentre, IL) from five infected and five healthy (control) plants. Strand-specific RNA-seq libraries were generated by the UC Davis Genome Center DNA Technologies Core Facility from the ribo-depleted RNA samples using an Apollo 324 liquid handler (Wafergen, CA) and PrepX RNA library preparation kits (Wafergen, CA) following the instructions of the manufacturer. After a cleanup step using 1x volume of Ampure XP beads (Beckman Coulter, CA), the single-end RNA-seq libraries were PCR-amplified using Phusion High-Fidelity polymerase (NEB, MA) following standard procedures, cleaned up again using a 1x volume of Ampure XP beads, and then quantified by fluorometry (Qubit; LifeTechnologies, CA). Libraries were analyzed with a Bioanalyzer 2100 instrument (Agilent, CA) and then pooled in equimolar ratios according to the fluorometric measurements. The pooled libraries (from five infected plants and five non-infected plants) were quantified by qPCR with a Kapa Library Quant kit (Kapa, South Africa) and sequenced on one lane of an Illumina HighSeq 2500 (Illumina, CA).

### Transcriptome Data Analysis and Validation

RNA-seq read quality and contamination were assessed with FastQC v.0-.10.1^1^. Scythe v.0.991 and Sickle v.1.210^2^ were used for Illumina adapter and quality-based trimming, respectively. Reads trimmed to less than 25 bases were discarded. *V. vinifera* genome assembly version IGGP 12x and annotation data used in this analysis can be found at Genoscope (Jaillon et al., 2007) and Ensembl Gramene release 51 (Tello-Ruiz et al., 2016).

Reads were aligned to the *V. vinifera* genome using bowtie2 v.2.1.0 (Langmead and Salzberg, 2012). A reference transcriptome was generated from the NCBI files, using the gffread program within cufflinks v.2.1.1 (Roberts et al., 2011). BWA’s short read aligner v.0.6.2 (Ardales et al., 2009) was then used to align the reads to the augmented transcriptome. Raw counts per gene were generated from the bwa alignments using sam2counts.py^3^. The raw counts from each of the five diseased and five control samples were statistically analyzed with EdgeR (Robinson et al., 2010) to produce tables of expression values, fold changes, and selection of differentially expressed (DE) transcripts, using *p* < 0.05 or 0.01 from the fitted negative binomial generalized linear models and quasi-likelihood F-test (Supplementary Tables S2–S5). Enrichment of gene ontology terms of DE transcripts was performed with PANTHER using Bonferroni correction and *p-*value cutoff of 0.05 (Mi et al., 2016). Reverse transcription – quantitative polymerase chain reaction (RT-qPCR) was performed as detailed in Dandekar et al. (2012) as a means to verify the expression data obtained with RNA-seq, encompassing genes with distinct overall expression levels and ratios between infected and non-infected samples, as well as distinct functional categories. A two-tailed paired sample Student’s *t*-test (alpha = 0.05) was performed with XLSTAT software on delta-Cq values to determine statistical significance of differences between infected and non-infected samples (three biological replicas assayed in duplicate each). One and two asterisks indicate, respectively, *p*-values < 0.05 and 0.01. Oligonucleotide primers used in RT-qPCR are listed in Supplementary Table S6. Transcriptome data have also been deposited in NCBI SRA under BioProject accession number PRJNA390670.

### Proteome Analysis

Protein extraction from 500 mg of ground grapevine leaf preparations (three diseased samples vs. three healthy controls) was performed with P-PER plant protein extraction reagent (Thermo Scientific). Samples were reconstituted in phosphate buffer saline (PBS) and 300 μg were precipitated with 4x volume of precipitation reagent (CalBiochem) according to manufacturer’s instructions. Precipitated samples were reconstituted in 100 μl of 6 M urea + 5 mM 1,4-dithiothreitol (DTT) and incubated at 37°C for 30 min; 15 mM iodoacetoamide (IAA) was added and incubated at room temperature for 30 min. IAA was then quenched with 30 mM DTT and incubated for 10 min. Lys-C/trypsin was added to a 1:25 enzyme:protein ratio and incubated at 37°C for 4 h; 50 mM ammonium bicarbonate was added to dilute urea and activate trypsin and digestion occurred overnight at 37°C. Digested peptides were then de-salted using Aspire RP30 Desalting Tips (Thermo Scientific) and resuspended in loading buffer.

The digested peptides were analyzed using a QExactive mass spectrometer (Thermo Fisher Scientific) coupled with an Easy-LC (Thermo Fisher Scientific) and a nanospray ionization source. The peptides were loaded onto a trap (100 micron, C18 100 Å 5U) and desalted online before separation using a reverse phased column (75 micron, C18 200 Å 3U). The gradient duration for separation of peptides was 60 min using 0.1% formic acid and 100% acetonitrile for solvents A and B, respectively. Data were acquired using a data-dependent ms/ms method, which had a full scan range of 300–1,600 Da and a resolution of 70,000. The ms/ms method’s resolution was 17,500 and an isolation width of 2 *m/z* with normalized collision energy of 27. The nanospray source was operated using 2.2 kV spray voltage and a heated transfer capillary temperature of 250°C. Raw data were analyzed using X!Tandem (Fenyo and Beavis, 2003) and visualized using Scaffold Proteome Software (version 4.4.1). A protein was considered identified when at least two peptides were mapped to it with >99% confidence threshold. Proteins with differential abundance between infected and non-infected samples were chosen by *p* < 0.05 obtained from the Mann–Whitney test. Samples were searched against Uniprot databases appended with the cRAP database, which contains common laboratory contaminants. Reverse decoy databases were also applied to the database prior to the X!Tandem searches. Raw and differential analysis data are presented in Supplementary Tables S7, S8. The proteome procedure was performed at the UC Davis Proteomics Core.

### Immunodetection of Proteins

Anti-ferritin HRP-conjugated polyclonal antibody was generated in rabbit by injecting synthetic peptides corresponding to structural epitopes (GenScript, NJ). Antibody was diluted in PBS-M 1% (PBS plus 1% non-fat dried milk) at a 1:500 dilution. Blocking and washing used PBS-M 5% (PBS plus 5% non-fat dried milk) and PBS-T 0.1% (PBS plus 0.1% Tween 20), respectively, and blots were developed using ECL Plus Western Blotting Detection Reagents (GE Life Sciences, United States) and visualized using a ChemiDoc-It TS2 (BioRad, CA) imaging instrument.

### Metabolome Analysis

Metabolomic analysis was performed at the NIH West Coast Metabolomics Center, UC Davis. Sample preparation and gas chromatography coupled to time-of-flight mass spectrometry (GC-TOF/MS) followed the protocols in Sana et al. (2010), including data filtering, BinBase assignment, and statistics. Eight samples from diseased plants vs. eight control healthy samples were compared. Metabolite extraction was done on 100 mg of ground leaf tissue extracted for 20 min at -20°C in pre-cooled 2:3:3 v/v/v solvent mixture of water/acetonitrile/isopropanol and centrifuged 16,000 *g* for 3 min. The liquid phase supernatant was used for GC-TOF/MS performed on an Agilent 6890 gas chromatograph (Santa Clara, CA, United States) controlled by the Leco ChromaTOF software vs. 2.32 (St. Joseph, MI, United States). A 30 m long, 0.25 mm internal diameter rtx5Sil-MS column with 0.25 μm 5% diphenyl/95% dimethyl polysiloxane film and additional 10 m integrated guard column was used (Restek, Bellefonte, PA, United States). Absolute spectra intensities were processed by a filtering algorithm implemented in the metabolomics BinBase database (Fiehn et al., 2005). Quantification was reported as peak height using the unique ion as default, unless a different quantification ion was manually set in the BinBase administration software Bellerophon. Metabolites were unambiguously assigned by the BinBase identifier numbers, using retention index and mass spectrum as the two most important identification criteria. Additional confidence criteria were given by mass spectral metadata, using the combination of unique ions, apex ions, peak purity, and signal/noise ratios as given in data preprocessing. All database entries in BinBase were matched against the Fiehn mass spectral library of 1,200 authentic metabolite spectra and the NIST05 commercial library (Kind et al., 2009). BinBase entries were named manually by both matching mass spectra and retention index. Statistical evaluation was performed using univariate Student’s *t*-test for independent pairs of groups. Values of *p* < 0.05 were considered statistically significant. Raw data and differential analysis are presented in Supplementary Tables S9, S10. Multivariate statistical analysis was performed with XLSTAT software by considering the Pearson correlation among the samples and plotted using Circos (Krzywinski et al., 2009).

## Results

### Multiomic Analysis of Pierce’s Disease Delineates the Pathogen Perception and Host Response Circuitry

**FIGURE 2:**
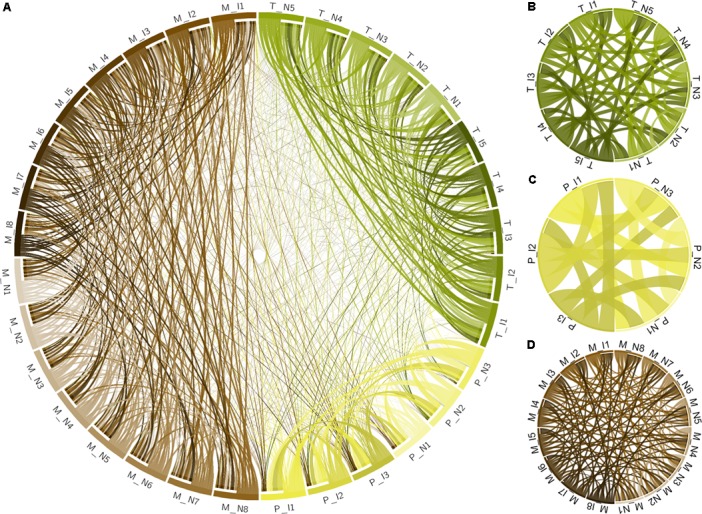
Multivariate analysis of datasets used in this work. **(A)** Circos plot of correlation matrix of all 32 samples, including infected (I) and non-infected (N), transcriptome (T), proteome (P), and metabolome (M) samples. Samples are represented by segments on the circumference and ribbons connecting samples indicate the relative contribution of each interaction to the total of that sample. Interactions only among the 10 transcriptome samples **(B)**, 6 proteome samples **(C)**, and 16 metabolome samples **(D)** are also shown. Correlation matrix is shown in Supplementary Table S1.

**FIGURE 3:**
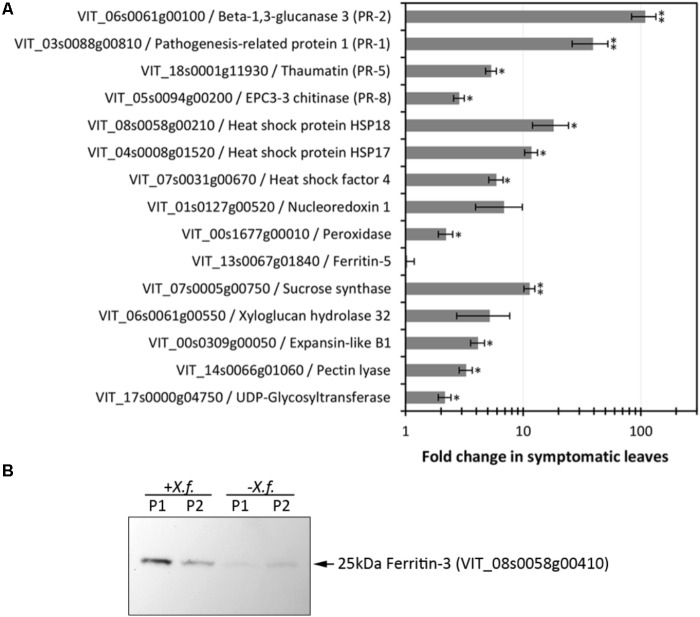
Evaluation of expression levels of selected CDS and proteins. **(A)** RT-qPCR analysis of CDS selected based on RNA-seq data encompassing various functions and modulation intensities in samples from diseased leaves. Fold changes compared to healthy control plants were calculated from three biological replicas assayed in triplicate each. One and two asterisks indicate, respectively, *p*-values < 0.05 and 0.01, according to two-tailed *t*-test for two paired samples with alpha = 0.05. **(B)** Immunodetection of ferritin 3 in leaf samples (P1, P2: two plants per treatment) infected with *X. fastidiosa* (*Xf*).

*Xylella fastidiosa*-infected grapevines under controlled greenhouse conditions started to show initial PD symptoms on leaves and stems ∼8 weeks post inoculation. These were initially limited to scorching of leaf blades near margins, progressing inward toward petioles. By the time, our samples were collected 12 weeks post inoculation, brownish patches were already visible on stems, and some leaves had already dropped off leaving typical “matchstick” petioles and an overall dehydrated appearance (**Figures 1A,B**). Transversal cuts of stems near the apical meristem showed that besides the brownish patches on canes, intensive alterations were also occurring inside the stems, marked by an increase in secondary cell wall deposition and thickening on infected vines (**Figure 1C**). Under these conditions, the leaf samples were collected and processed for RNA-seq deep sequencing, isobaric labeling proteome mass spectrometry, and GS-TOF/MS metabolite profiling. With all three methodologies, we were able to identify analytes over- and under-represented in symptomatic vines, both of known and unknown functions (**Table 1**). Multivariate analysis was used to determine the correlation among the datasets used in this work (**Figure 2**), which shows as expected that experimental technique (transcriptome, proteome, or metabolome) is a stronger determinant of higher correlation than experimental group (infected or non-infected). Considering only transcripts with low variability among replicas (*p* < 0.05), we found only 16 genes that we also detected as DE in the proteome dataset. These encompass ATP synthase subunit alpha (VIT_00s0733g00010), ADP/ATP carrier (VIT_08s0007g02450), HtpG chaperone family protein (VIT_02s0025g04340), dehydrin (VIT_04s0023g02480), lipid-transfer protein (VIT_05s0020g03750), beta 1,3-glucanase 3 (VIT_06s0061g00120), alpha-beta hydrolase (VIT_07s0005g01240), ferritin 3 (VIT_08s0058g00410), thioredoxin superfamily protein (VIT_12s0028g03010), chitinase-18 (VIT_14s0066g00610), subtilisin-like proteins (VIT_15s0048g01180, VIT_18s0001g14870), PHB domain-containing membrane-associated protein (VIT_16s0100g00090), MYB4 (VIT_17s0000g04750), glycine-rich RNA-binding protein (VIT_18s0001g11930), and a Clp protease (VIT_19s0014g03160). Although these overlapping results from the transcriptome and proteome datasets illustrate fragments of the host response to infection, many other details were captured in each dataset and will be presented next. A complete dataset of detected metabolites, including their relative levels compared to those in healthy vines, are available as Supplementary Materials, and grouped by transcriptomic, proteomic, and metabolomic data (Supplementary Tables S1–S11). Among the methods employed in this work, our RNA-seq dataset is the most comprehensive and revealed expression for 23,991 unique protein coding sequences (CDS), representing 80% of the 29,971 CDS predicted in the CRIBI V1 grape genome annotation. To further validate the transcriptome data, we performed RT-qPCR on 15 CDS from *X. fastidiosa*-infected leaf extracts encompassing a wide range of response intensities (**Figure 3A**) and western blot of ferritin (**Figure 3B**). These additional techniques also show that while some ferritins respond to disease, other paralogs do not, as previously suggested by the transcriptome and proteome data. More details on the ferritins will be presented ahead.

**Table 1:** Summary of multiomics data from grapevine leaves with Pierce’s disease.

*Transcriptome*	Increased^a^	Decreased	Total
CDS with putative function	854	238	19140
CDS without putative function	123	25	4907
Total detected	977	263	23991
*Proteome*			
Proteins with putative function	20	15	98
Proteins without putative function	69	23	263
Total detected	89	38	361
*Metabolome*			
Identified metabolites	14	4	121
Unidentified metabolites	29	3	230
Total detected	43	7	351

*^a^Increased and decreased abundance in diseased leaves compared to healthy controls.*

**FIGURE 4:**
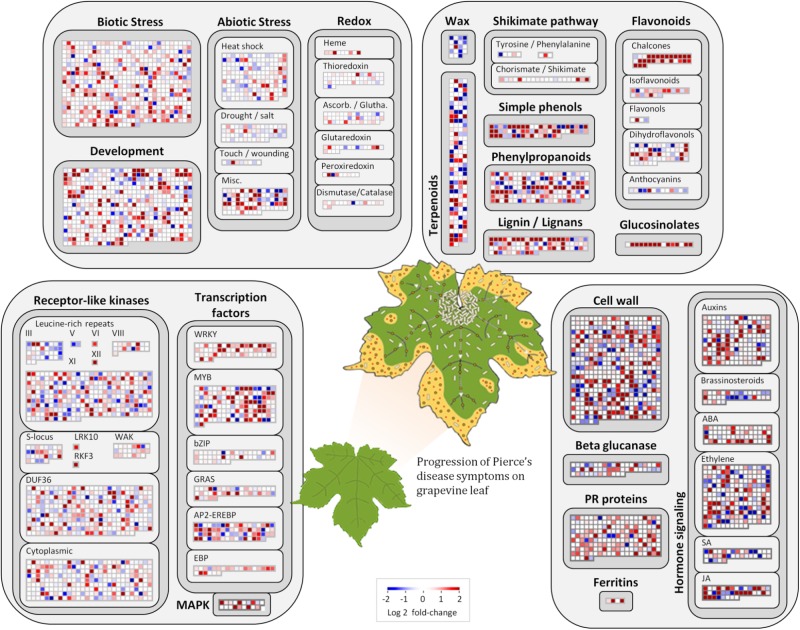
Molecular functions affected by Pierce’s disease. Comparative transcriptome data grouped by functional categories are available in MapMan. Color scale represents log2 ratios of diseased vs. healthy grapevine leaves, in which each protein coding sequence is represented by a square within a selected functional category. Leaf diagram represents the transition of from healthy to diseased tissues analyzed in this comparison. Pale rods on leaf represent *X. fastidiosa* cells occurring within xylem vessels, at higher density near the petiole, and gradually decreasing toward margin, where outer membrane vesicles and secreted proteins are still abundant (*n* = 5 diseased vs. 5 healthy plants).

**FIGURE 5:**
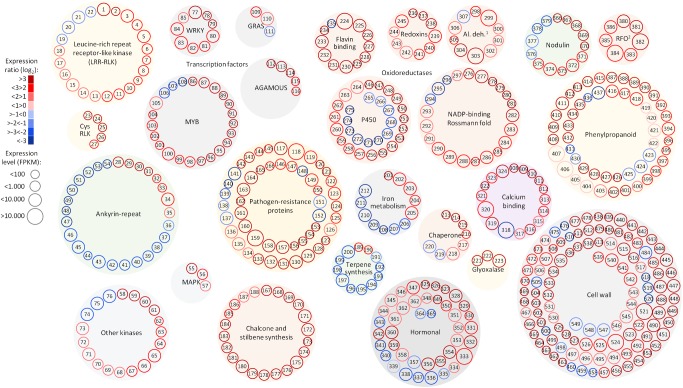
Selected functional categories responsive to Pierce’s disease. Representation showing expression ratio and level of transcripts with altered abundance in grapevines leaves showing Pierce’s disease symptoms mentioned in this work. Log_2_ expression ratios were calculated from averages of five symptomatic samples compared to five healthy samples, with padj < 0.05. Normalized expression levels are represented in reads per kilobase of transcript per million mapped fragments (FPKM), from the sum of average values of symptomatic and healthy samples. ^1^Al. deh. = alcohol and aldehyde dehydrogenases and ^2^RFO = raffinose family of oligosaccharides. A complete list of genes and values shown in this figure is given in Supplementary Table S11.

Among the 1,240 DE CDS, 47 were detected only in infected tissues including laccases, polygalacturonase, pectin lyase, and fasciclin-like arabinogalactan protein (FLA) genes mainly involved in cell-wall remodeling and lignification (Wang et al., 2015). A gene ontology analysis of all DE CDS shows enriched terms describing biological processes and molecular functions of these and other DE genes, as listed in **Table 2**. Clustering of transcripts by sequence similarity also reveals enriched functions in the transcriptome data, such as chalcone and stilbene synthases, laccases, and other cell wall remodeling enzymes (Supplementary Table S5). The systems approach was further extended to MapMan functional analysis (Thimm et al., 2004), revealing various aspects of the metabolic shift accompanying PD development, from perception of the pathogen to cell wall modification, onset of oxidative stress response, and polyphenol metabolism to counteract chronic oxidative damage (**Figure 4**). Although convenient to visualize fold-changes of specific genes of major metabolic functions affected by the disease, we also sought to identify robust disease markers by including the notion of expression level of each marker, as shown in **Figure 5**. Taken together, these parameters of relative abundance provide a wealth of information describing the molecular events that will be explored in the following sections.

**Table 2:** Summary of PANTHER statistical overrepresentation test results for enrichment of GO terms in differentially expressed genes.

GO term	Term description	Fold enrichment	*p*-value	Transcript abundance
*Biological process*				
GO:0009698	Phenylpropanoid metabolic process	5.85	1.15E-03	+
GO:0009813	Flavonoid biosynthetic process	5.23	1.59E-03	+
GO:0046942	Carboxylic acid transport	4.95	1.64E-02	+
GO:0016052	Carbohydrate catabolic process	3.56	3.41E-02	+
GO:0032787	Monocarboxylic acid metabolic process	2.91	1.60E-03	+
GO:0071554	Cell wall organization or biogenesis	2.68	3.53E-03	+
GO:0055114	Oxidation–reduction process	1.77	4.64E-02	+
GO:2000031	Regulation of salicylic acid mediated signaling pathway	50.42	1.30E-04	-
GO:0016042	Lipid catabolic process	7.47	8.05E-03	-
*Molecular function*				
GO:0035251	UDP-glucosyltransferase activity	4.73	3.68E-02	+
GO:0048037	Cofactor binding	3.23	2.25E-04	+
GO:0022857	Transmembrane transporter activity	2.16	8.22E-03	+
GO:0016491	Oxidoreductase activity	1.84	6.43E-03	+
GO:0043167	Ion binding	1.35	4.73E-02	+
GO:0052689	Carboxylic ester hydrolase activity	6.44	1.83E-03	-
GO:0016705	Incorporation or reduction of molecular oxygen	4.82	2.71E-02	-
*Cellular component*				
GO:0071944	Cell periphery	1.64	2.29E-05	+
GO:0005886	Plasma membrane	1.58	1.78E-03	+
GO:0016021	Integral component of membrane	1.45	3.54E-04	+
GO:0005576	Extracellular region	2.45	6.87E-05	-

**FIGURE 6:**
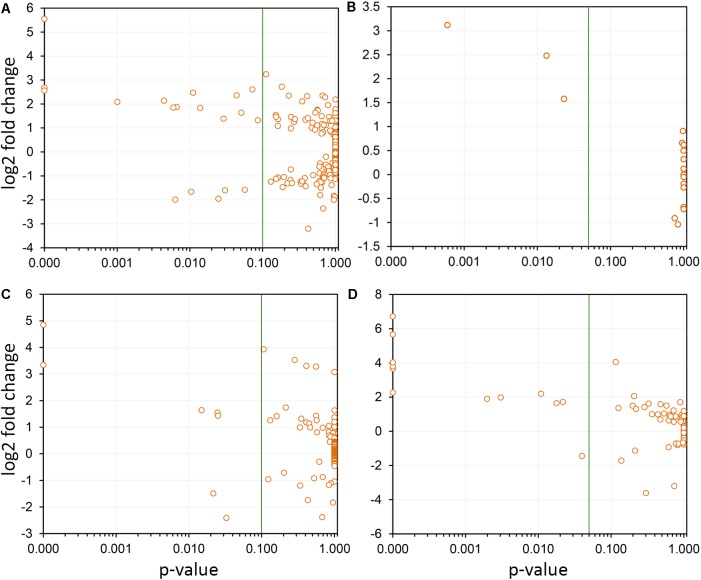
Selection of paralogs responsive to Pierce’s disease. Four examples are shown, in which the members of paralogous groups that are considered responsive to disease can be selected based on confidence levels (variability among replicas). In each panel, the green line indicates the *p*-value of 0.05 selection threshold used by us. **(A)** A total of 291 leucine-rich repeats receptor-like kinases were detected as expressed, and while only 19 were considered responsive to disease, 14 showed increased transcript abundance, and 5 reduced. **(B)** Seventeen thaumatin-like genes were detected as expressed, being three with increased transcript abundance. **(C)** From the 122 2-oxoglutarate oxygenase paralogs detected as expressed, seven were responsive to disease, with five showing increased transcript abundance. **(D)** From the 74 cellulose synthases detected as expressed, 12 were responsive to disease and of these only one shows reduced transcript abundance.

Differentially expressed CDS related to extracellular signal perception including 22 leucine-rich repeat (LRR) receptor-like kinases (RLKs) were highlighted, with emphasis to VIT_14s0128g00550 and VIT_10s0003g01430. We used the genes encoding LRR-RLKs as an example of the power of genome-wide investigations to select the paralogous members within the functional family group that are relevant to PD (**Figure 6A**). Transcripts encoding five cysteine-rich RLKs also showed increased abundance (VIT_00s0262g00120, for example), indicating a multitude of input signal sources. CDS encoding an array of ankyrin-repeat proteins were also strongly modulated, especially VIT_13s0106g00200 positively and VIT_05s0165g00260 negatively. Signal transduction cascade MAPKs were also upregulated (VIT_06s0004g06850, VIT_07s0031g00530, and VIT_12s0059g00870) plus many other cytosolic kinases of various types. We also detected WRKY9 (VIT_12s0055g00340), MYB108 (VIT_05s0077g00500), GRAS (VIT_13s0019g01700), and an AGAMOUS-like (VIT_17s0000g01230) transcription factors (TFs) as the most responsive to PD, among many others modulated less intensively (Supplementary Table S4). Another interesting group of DE transcripts encode members of four of the seven families of nodulin-like proteins, including seven MtN21/UMAMIT-like (VIT_04s0044g00450), five MtN3/SWEET-like (VIT_11s0016g04920), two early nodulin-like (VIT_14s0066g01420), and three vacuolar iron transporter/nodulin-like (VIT_00s0267g00030).

### Pathogenesis-Related Proteins and Antimicrobial Compounds

Among the responses to *X. fastidiosa* infection, we found accumulation of transcripts encoding pathogenesis-related (PR) proteins, which constitute a complex repertoire of defense strategies aimed at inhibiting pathogen proliferation (Sels et al., 2008). These encompassed several β-1,3-glucanases (for example, VIT_05s0077g01150), class I, II, III, and V chitinases (PR-11, PR-4, PR-8, PR-3, with VIT_14s0066g00610 most intensively modulated), thaumatin-like proteins (PR-5, such as VIT_18s0001g14480; **Figure 6B**), proteinase inhibitors (PR-6, VIT_05s0020g05040), proteinases (PR-7, VIT_07s0104g00180), peroxidases (PR-8, VIT_14s0066g01670), ribonucleases (PR-9, VIT_14s0060g01530), lipid transfer proteins (PR-14, VIT_06s0004g08060 upregulated and VIT_08s0058g01210 downregulated), and oxalate oxidase germins (PR-15 VIT_09s0002g01340, and PR-16 VIT_07s0005g02370). The proteome data also highlight the chitinase VIT_14s0066g00610 as one of the most modulated in infected tissues, corroborating the transcriptome data. Among the PR proteins, 12 CDS encoding germin-like proteins from the RmlC-like cupins superfamily were upregulated, with VIT_14s0128g00600 in greater intensity. Three Kunitz-type protease inhibitors were also strongly induced (VIT_17s0119g00150, among others).

**FIGURE 7:**
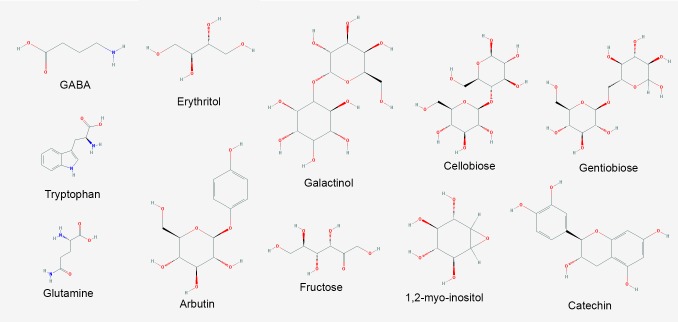
Major metabolites with increased abundance in *V. vinifera* affected by Pierce’s disease. PubChem structures of metabolites with known antimicrobial and signaling functions accumulated in symptomatic grapevines (*n* = 8 diseased vs. 8 healthy plants).

Specialized (secondary) metabolism was also strongly influenced by disease onset. Twenty-two upregulated CDS for chalcone and stilbene synthases, involved in phytoalexin production against microbes (Aoki et al., 2016), and plant defense against abiotic stress such as UV-radiation were highlighted in our dataset, with VIT_16s0100g01040 most intensively, and others in the same genomic vicinity of chromosome 16. Terpene metabolism was also modified according to our data, with increased abundance of transcripts for terpene synthases VIT_18s0001g04120 and VIT_00s0692g00020, while reducing that of 10 others with VIT_19s0014g04930 and VIT_00s0271g00010 most intensively. Enrichment of compounds known to inhibit pathogen growth and biofilm formation was also detected in our metabolome data such as erythritol and 2-deoxyerythritol (Ghezelbash et al., 2012), 1,2-anhydro-myo-inositol and arbutin; these latter glycosidase and tyrosinase inhibitors, respectively (Falshaw et al., 2000; Supplementary Table S10 and **Figure 7**). The pathogen might also benefit from increased concentration of nutritional compounds such as fructose, tryptophan, and glutamine.

### Oxidative, Drought, and Osmolarity Stress-Related Responses

Iron sequestration was triggered by disease onset, with ferritin isoforms VIT_08s0058g00410, VIT_08s0058g00430, and VIT_08s0058g00440, being strongly induced, along with aconitase (VIT_12s0059g02150) and nicotianamine synthase 4 (VIT_11s0052g01150), involved in phytosiderophore production (Mizuno et al., 2003). Interestingly, these three ferritins encoded in chromosome 8 were intensively upregulated, while another present on chromosome 6 (VIT_06s0004g07160) was not selected based on expression ratio, but remained highly expressed also in healthy samples. Another ferritin on chromosome 13 (VIT_13s0067g01840) had very low expression in grapevine leaves with or without disease. Three vacuolar iron transporters on the other hand were downregulated (most markedly VIT_00s0267g00030). Modulation of other iron-associated redox proteins such as ferric reductase, iron superoxide dismutase, and ferredoxin were not significant. Besides limiting iron availability to microbes, storing free iron within ferritin nanocages reduces ROS production and oxidative damage (Mehterov et al., 2012). Like in the transcriptome data, ferritin-3 (VIT_08s0058g00410) was intensively upregulated in the proteome data and further confirmed by western blot (**Figure 3B**), emerging as a robust feature of PD onset.

Other intense responses included upregulation of proteins and transcripts for low molecular weight heat shock proteins, glyoxalase I for methylglyoxal detoxification (for example, VIT_09s0002g06430), and anthocyanidin reductases such as VIT_15s0046g01150 involved in general stress tolerance (Xie et al., 2003; Hasanuzzaman et al., 2017). Anthocyanins evidenced in symptomatic leaves (**Figure 1B**) might come from the upregulated biosynthetic pathway encompassing phenylalanine ammonia-lyase (VIT_11s0016g01640 and other copies), cinnamate-4-hydroxylase (VIT_11s0078g00290), 4-coumarate CoA ligase (VIT_06s0061g00450), chalcone synthase (VIT_16s0100g01040 plus several others in this genomic vicinity), chalcone isomerase (VIT_04s0008g02030), flavanone 3-hydroxylase (VIT_18s0001g14310), leucoanthocyanidin dioxygenase (VIT_02s0025g04720), UDP-glucosyl transferase (VIT_18s0001g12040 and others), and finally methyltransferases (VIT_16s0100g00570 and others), possibly providing an array of different anthocyanins yet to be further characterized. Moreover, CDS for 21 NADP-binding Rossman-fold enzymes with oxidoreductase activity were modulated, with VIT_15s0046g01150 as the most prominent. Concurrent to this, our proteome data also showed photosynthesis components such as photosystem I and II, electron transport, carbon fixation, and ATP synthesis with decreased abundance, which could also contribute to a reduction of chlorophyll levels unmasking anthocyanins already present.

The metabolite with highest increased abundance detected on symptomatic leaves was gamma-aminobutyric acid (GABA), a known response for water, oxidative, and wounding stress in *Arabidopsis thaliana* (Kinnersley and Turano, 2000; Coleman et al., 2001; Scholz et al., 2017). On the other hand, its precursor, glutamic acid, showed decreased abundance, supporting this intense metabolic flow (Supplementary Table S10 and **Figure 7**). These metabolome data are consistent with upregulation of two CDS encoding glutamate decarboxylase (GAD) in our transcriptome data (VIT_01s0011g06600 and VIT_01s0011g06610), which perform this enzymatic conversion. Our metabolome data also indicate several sugars upregulated in infected tissues, besides other metabolites with known antioxidant properties, including galactinol, catechin, cellobiose, and gentiobiose. These metabolites follow a trend observed in the transcriptome and proteome data in which several ROS-scavenging systems were also upregulated, such as three paralogs of galactinol synthase (VIT_05s0077g00430 among others) and a raffinose hydrolase known as seed imbibition 2 (VIT_08s0007g08310) which also helps to increase galactinol levels in response to pathogen attack and oxidative stress (Couee et al., 2006; Sengupta et al., 2015). Two other raffinose synthase family protein CDS (VIT_11s0016g05770 and VIT_07s0005g01680) were also highly upregulated possibly providing the substrate for galactinol synthesis. We also detected modulation of various cytochrome P450s with emphasis to VIT_07s0129g00820, besides seven peroxidases (six with increased abundance lead by VIT_14s0066g01670 and VIT_18s0001g06840). Other modulated enzymes involved in ROS turnover include five alcohol and five aldehyde dehydrogenases (VIT_18s0001g15410 and VIT_01s0026g00220, for example), glutathione S-transferases including VIT_04s0079g00690 and Tau7-like VIT_16s0039g01070, and quinone reductases encoded by VIT_00s0271g00110 and VIT_00s0274g00080, all of these playing a role in ROS turnover and byproduct detoxification. Interestingly, however, while some enzymes such as cysteine peroxiredoxin (VIT_05s0020g00600) and thioredoxin (VIT_12s0028g03010) accumulated both in the transcriptome and proteome data, other classical ROS-detoxifying enzymes such as superoxide dismutase, catalase and glutathione peroxidase did not. Other DE transcripts involved in ROS signaling also include CDS that bind calcium such calmodulins (VIT_18s0122g00180 and five others), calcineurins (VIT_17s0000g09480 with increased abundance and VIT_17s0000g09470 with a sharp decrease), Ca^2+^-binding EF-hand family proteins (VIT_12s0059g00340 and two others), as well as Ca^2+^-dependent lipid-binding (CaLB domain) family proteins. The EF-hand proteins are known to activate respiratory burst oxidase homologs (RBOH, VIT_14s0060g02320 also upregulated in our data), capable of ROS production upon pathogen perception (Kadota et al., 2015). Moreover, three CDS encoding lactoylglutathione lyase/glyoxalase I (methyglyoxal detoxification), which are also calcium binding proteins are among the most induced in our dataset, particularly VIT_11s0016g05010. Many of the responses to oxidative stress listed above are also detected in other kinds of stresses such as drought and osmolarity, exemplifying how PD results in various stresses to the plant host. All these responses interconnect resulting in the scorched leaves and other symptoms observed in diseased vines as shown in **Figure 1**. Another interesting connection between different stresses is major facilitator superfamily proteins for which eight CDS including VIT_05s0020g02170 were upregulated and two were downregulated. Besides responding to salt stress, these sugar/H+ symporters have being shown to be tightly correlated with programmed cell death (Norholm et al., 2006), as has flavin-dependent monooxygenases encoded by VIT_11s0016g00570 and VIT_07s0104g01260, also with increased abundance in diseased vines.

### Modulation of Hormone Biosynthesis and Signaling

Jasmonate biosynthesis was a strong hormonal response detected given the intensive positive regulation of seven 12-oxophytodienoate reductases including VIT_18s0041g02020 and two allene oxide synthases (VIT_03s0063g01820 and VIT_18s0001g11630), known routes for jasmonic acid formation (Schaller et al., 2000). However, transcripts encoding downstream jasmonate *O*-methyltransferases (VIT_04s0023g02230 among three other paralogs) and other DE CDS encoding enzymes of salicylic acid formation displayed a strong reduction in abundance. Despite the suggested inhibition of salicylic acid production, transcripts encoding the AGD2-like defense response protein 1 (*ALD1*, VIT_18s0001g04630), accumulated to higher levels in diseased vines. On the catabolic side, the methyl salicylate esterase VIT_00s0253g00140 was upregulated, possibly also contributing to lowered accumulation of salicylic acid. Gibberellin (GA) production also seems repressed as indicated by downregulation of 2 GA oxidases (VIT_18s0001g01390 and VIT_19s0177g00030), a MEP pathway member (VIT_11s0052g01730), and a gibberellin-regulated protein (VIT_08s0007g05860). Abscisic acid (ABA) biosynthesis epoxycarotenoid dioxygenase (VIT_19s0093g00550), and ABA-responsive protein (VIT_18s0001g10450), and two PP2C phosphatases (VIT_06s0004g05460 and VIT_16s0050g02680) were upregulated, another indication of the multiple stress physiology state of grapevines during PD. Although increased abundance of auxin biosynthesis enzymes was not detected, several auxin-responsive CDS were, including VIT_12s0057g00420, three auxin efflux carriers VIT_04s0044g01860, VIT_05s0062g01120, and VIT_04s0044g01880, auxin-induced protein VIT_05s0049g01970, auxin-responsive of the GH3 type (VIT_19s0014g04690, among three others), and TFs (VIT_11s0016g04490, VIT_18s0001g13930). We also detected 2-oxoglutarate and Fe^2+^-dependent oxygenase superfamily proteins with members both up and downregulated (Supplementary Table S4 and **Figure 6C**), which catalyze the formation of hormones, such as ethylene, gibberellins, and pigments such as anthocyanidins and other flavones (Turnbull et al., 2004), again illustrating the broad spectrum and interconnectivity of responses observed.

### Modulation of Functions Involved in Cell Wall Remodeling

Since the thickening of secondary walls of xylem vessels is one of the most striking anatomical changes during PD onset (**Figure 1C**), we analyzed various host responses that could be classified as related to cell wall metabolism. We detected several molecular systems working in cohort to produce the conspicuous thickening of xylem vessel walls. Some of the aforementioned enzymes of the phenylpropanoid biosynthesis pathways involved in the early steps of anthocyanin synthesis also generate precursors of monolignols. These are further processed and polymerized by oxidative enzymes, including peroxidases and copper-binding laccase-like polyphenol oxidases. Many laccases are highlighted in our data, with emphasis to VIT_18s0001g00680 and VIT_18s0122g00690 among 19 others, as well as six peroxidases including VIT_14s0066g01670. Transcripts encoding 6 FAD-binding Berberine family proteins involved in monolignol oxidation were also accumulated, as well as for cytochrome P450-dependent monooxygenases known to be involved in lignin biosynthesis (Daniel et al., 2015), including VIT_11s0078g00290 and VIT_11s0065g00350 (cinnamate-4-hydroxylase) and also VIT_04s0023g02900 (ferulic acid 5-hydroxylase).

Among other enzymatic functions involved with cell wall modification, we found reduced levels of two neighboring polygalacturonases (VIT_01s0127g00850 and VIT_01s0127g00870) and increased levels of a polygalacturonase inhibitor (VIT_08s0007g07690) and of five dehiscence zone polygalacturonases orthologs of AT2G41850, eight pectin lyases including VIT_07s0005g05520, 7 pectin invertase/methylesterase inhibitors such as VIT_06s0009g02590, 11 cellulose synthases with emphasis to VIT_02s0025g01940 (**Figure 6D**), the xyloglucan endotransglucosylase/ hydrolase VIT_06s0061g00550, and two uclacyanins, all established in cell wall modification and/or lignin biosynthesis (Minic et al., 2009). Our proteomic analysis also identified upregulated gluconeogenic enzymes fructose-bisphosphate aldolase and alpha-glucan water dikinase, as well as TCA cycle enzymes citrate synthase and isocitrate dehydrogenase, possibly contributing to generation of carbon backbones for cell wall thickening and ROS detoxification (Supplementary Table S8). Another protein with increased abundance was cinnamoyl-CoA reductase 1, known to participate in lignin biosynthesis (Ruel et al., 2009). Other modulated functions involved in cell wall modification functions encompassed eight expansins including VIT_13s0067g02930, the exostosin VIT_06s0061g00560, the beta galactosidase VIT_11s0016g02200, five beta glucosidases both positively and negatively regulated (VIT_07s0005g00360 and VIT_13s0064g01660, for example), two beta xylosidases (VIT_05s0077g01280), three beta 1,3-glucanases including VIT_06s0061g00100, and four hydroxyproline-rich LEA proteins (VIT_04s0069g01010 in greater abundance). DE transcripts also include 18 UDP-glucosyl transferases with emphasis to VIT_06s0004g07230, UDP-glucosyl epimerase VIT_02s0025g04210, and the UDP-glucosyl dehydrogenase VIT_17s0000g06960, all involved in callose formation and deposition (Ellinger and Voigt, 2014).

## Discussion

Much of our understanding of the plant responses to bacterial pathogens has been interpreted by the gene-for-gene response mediated by the secretion of type 3 effectors and their interaction with host R-proteins as a means to identify sources of resistance genes. Yet *X. fastidiosa* is a very successful pathogen for many economically important crops despite lacking a type 3 secretion system (T3SS) and other elicitors of plant immunity such as flagella. It uses, however, a T2SS that has been shown to secrete a number of hydrolytic enzymes that correlate to the observed disease symptoms. Our data further depict a wealth of molecular details of its complex interaction with grapevines leading to PD that suggest: (1) activation of a complex defense response that includes both pathogen- and damage-associated molecular pattern (PAMP/DAMP)-triggered immunity (PTI), but impairment of downstream salicylic acid-mediated immune response; (2) chronic oxidative stress despite activation of antioxidant metabolism; and (3) intensive cell wall remodeling and lignification, which can lead to reduced sap flow and increased water and nutrient deficiency. This is an expansion from the previous transcriptome investigation of the early events of PD development performed by Choi et al. (2013), in which the molecular events leading to water stress were also detected.

Given the xylem-dwelling characteristic of the pathogen, deep within the plant, and its ability to form biofilms and secrete virulence factors, pathogen clearance is not achieved in susceptible hosts despite the array of responses observed, including PR proteins and phytoalexins. Our data expand the set of PR proteins previously detected in the xylem sap of *X. fastidiosa*-infected grapevines (Chakraborty et al., 2016) further reinforcing their importance in the defense response. Though fastidious in growth, *X. fastidiosa* can actively migrate with and against xylem sap flow (Meng et al., 2005) and colonize the plant systemically, overcoming host strategies to halt pathogen proliferation. Callose and tylose formation are commonly seen throughout affected branches, and symptoms on leaves are not always associated with *X. fastidiosa* presence on the vicinity of scorched areas, suggesting the recognition and response to secreted effectors, PAMPs, and/or DAMPs (Gambetta et al., 2007; Nascimento et al., 2016; Rapicavoli et al., 2018). These signals might be associated to long range outer membrane vesicles, bacterial molecular signals, or products of host tissue degradation (Lindow et al., 2014; Nascimento et al., 2016; Rapicavoli et al., 2018). Pathogen and damage perception is suggested by induction of many LRR-RLKs, as well as downstream MAPKs and TFs of WRKY, and other types, all important for PTI (Banerjee and Roychoudhury, 2015). These receptors are the first layer of pathogen perception on the plant cell surface and directly or indirectly activate MAPK signaling cascades that affect a large group of TFs controlling the response circuitry (Zipfel, 2014). A large group of CDS encoding a wide array of ankyrin-repeat proteins were also strongly modulated, which have been suggested to be intermediates between membrane-kinase receptors and downstream MAPKs (Yang et al., 2012), and were shown to increase resistance against bacterial blight caused by *Xanthomonas oryzae* (Wang et al., 2006) among other examples. It would be interesting to identify specific elicitors and signaling molecules recognized as PAMPs in *X. fastidiosa* pathosystems, and their cognate receptor pairs, as done, for example, between grapevine and *Burkholderia phytofirmans* (Trouvelot et al., 2014). In the case of *Xylella* that lacks flagella, this might help the pathogen to reduce the amount of immunogenic epitopes, as shown in other Xanthomonads that can regulate their flagellin biosynthesis, express multiple flagellin types, shed, or completely lack flagella (Darrasse et al., 2013), affecting their immunogenicity. We are currently pursuing this by expressing candidate *Xylella* proteins in grapevines, in order to better understand the host response to specific elicitors and assess whether PD symptoms derive from host responses to pathogen perception or from activity of the pathogen’s effectors. Another interesting aspect of this plant-microbe crosstalk is the strong induction of GABA. Besides being part of the host response, it can also be used by bacterial quorum sensing, as exemplified by *Agrobacterium tumefaciens* in tobacco (Chevrot et al., 2006). Its precise effect on *X. fastidiosa* cellular behavior remains to be verified.

Our work also suggests that grapevines susceptible to PD go through intensive oxidative stress during disease development, as sources of ROS from PTI and also from photooxidative stress were evident in our data. ROS-generating systems were activated, such as germins, cupins, RBOH, and copper amine oxidases, possibly as a means to restrict pathogen proliferation. On the other hand, chronic exposure to high ROS levels is also detrimental to the host, as suggested by several ROS-scavenging strategies activated, among which we highlight the iron-sequestration ferritin nanocages, phenylpropanoid biosynthesis pathways, plus other proteins, and many metabolites aforementioned. Iron is needed to produce chlorophyll (Kumar and Soll, 2000), and hence its persistent chelation and consequent deficiency might intensify the conspicuous chlorosis symptoms and reduction of photosynthetic activity associated with PD. Our work provides further detail of this complex crosstalk between responses to pathogen and oxidative and drought stresses, as previously investigated in *A. thaliana* (Huang et al., 2008).

The intensive secondary metabolism modulation occurring during disease progression involves phytoalexin pathways (stilbene synthases) and also more macroscopic features such as cell wall thickening by phenylpropanoid and lignin biosynthesis. Although these are known components of grapevine’s defense arsenal (Vannozzi et al., 2012), *X. fastidiosa* is able to evade these defense mechanisms and reach sufficient population thresholds that induce bacterial aggregation, biofilm formation, and efficient vector acquisition and transmission (Almeida et al., 2012). Exploring whether the upregulated metabolites can limit the bacterium’s ability to reach such thresholds might be a promising way for delaying of preventing vectored disease transmission. Other insights toward rational disease control can come from the characterization of host susceptibility genes that can be exploited by the pathogen to harness nutrients. The nodulins identified in this work are strong candidates in this path, as are the enzymes involved in salicylic acid metabolism that displayed decreased abundance possibly delaying immune responses. The sustained jasmonic acid-related responses coupled with inhibition of salicylic acid-related responses even after 12 weeks post-infection suggest that grapevines recognize *X. fastidiosa* as a necrotroph. Interestingly, this pattern of responses has also been observed in resistant citrus inoculated with *X. fastidiosa* during early stages of infection (1 day post-infection; Rodrigues et al., 2013). However, in the case of Thompson seedless grapevines, pathogen clearance is not attained and chronic exposure to oxidative stress combined with the virulence arsenal of the pathogen leads to plant death. Further investigations of particular pathways/analytes including more time-points will clarify the details and dynamics of the observed responses. As *Xylella* pathogens can switch between a more aggregated (biofilm) to a solitary/planktonic bioform, identifying the specific responses associated with each bioform is also warranted.

## Conclusion

**FIGURE 8:**
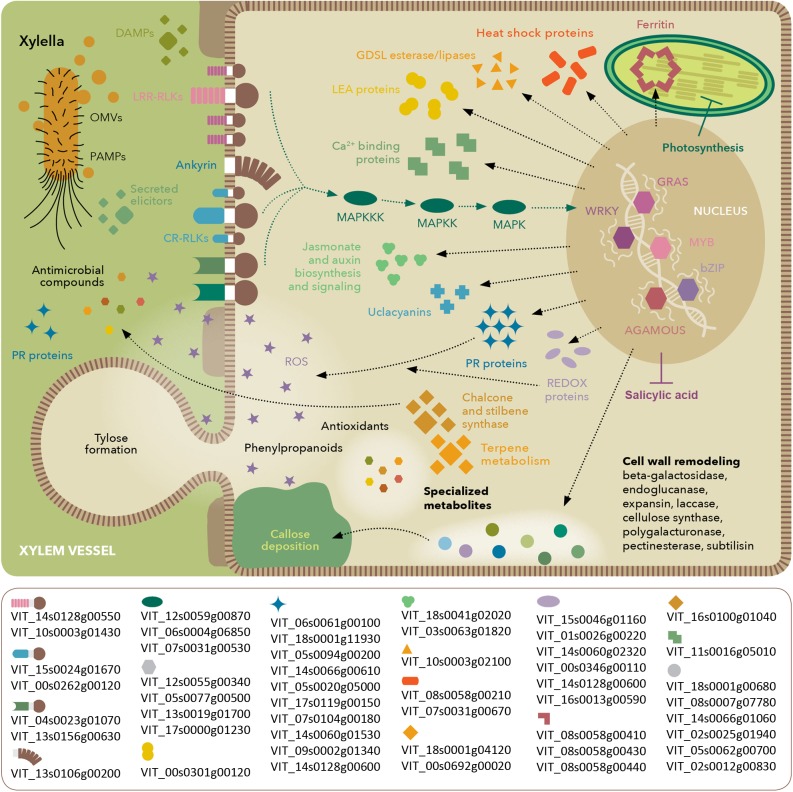
Model of molecular events occurring in *V. vinifera* with Pierce’s disease. Depiction based on transcriptomic, proteomic, and metabolomic analyses, highlighting perception of *X. fastidiosa* PAMPs and DAMPs, signaling cascades and stress response. The lower box lists prominent members in each functional category based on their expression ratios between diseased and healthy grapevines. Gene identifiers are based on the Ensembl Gramene release 51 *V. vinifera* annotation.

Our work provides a set of molecular markers modulated by PD onset, as detected by different omics strategies. Our work attests the usefulness of using different omics strategies to dissect a pathosystem. Although some general features are clear on all of them such as the oxidative stress the host is going through, many are only evident in one or two of the techniques used. Integrating the omics data still presents many challenges, such as uniform annotation and nomenclature used by different tools and databases. The very different depth of the data, such as between the transcriptome and the proteome, also limit the extent to which an integrated analysis can be done. Nonetheless, we see a trend toward greater depth on all omics approaches as equipment and protocols advance. At this stage, our data already enable the selection among the various paralogs of given functions, for example, among the LRR-RLKs, thaumatin-like proteins, 2-oxoglutarate oxygenases, and cellulose synthases, as shown in **Figures 5**, **6**. Understanding which paralogs respond most intensively to PD provides a valuable resource for targeted future investigations. The molecular markers for PD symptoms include proteins of various families plus antioxidant and antimicrobial metabolites that can now be further explored individually to evaluate their potential in disease detection and resistance. An integrated overview of the most intense responses detected is presented in **Figure 8**. Comparative studies with the available breeding germplasm, analyzing specifically the markers highlighted herein, and engineering specific enhanced or reduced gene functions in order to increase resistance or reduce detrimental immune responses by silencing susceptibility genes are promising ways to address this.

## Availability of Data

Raw transcriptome data are available for download at NCBI SRA BioProject # PRJNA390670. Filtered and normalized transcriptome data, along with proteome, metabolome data are available for download as supplementary material.

## Author Contributions

RN, DC, LG, and AD designed the research. PZ, RN, HG, MP, and SC performed the research, data analysis, and interpretation. PZ and AD wrote the manuscript. All authors read and approved the final manuscript.

## Conflict of Interest Statement

The authors declare that the research was conducted in the absence of any commercial or financial relationships that could be construed as a potential conflict of interest.

## Footnotes

1 http://www.bioinformatics.babraham.ac.uk/projects/fastqc/

2 https://github.com/ucdavis-bioinformatics

3 https://github.com/ucdavis-bioinformatics
